# Gamma-interferon assay for the ancillary diagnosis of bovine tuberculosis in dairy cattle in urban and adjacent areas of Dhaka city, Bangladesh

**DOI:** 10.14202/vetworld.2023.2120-2127

**Published:** 2023-10-17

**Authors:** Tanzida Begum Rumi, Sk. Shaheenur Islam, Robiul Islam, Md. Mahmudul Hasan Faisal, S. M. Lutful Kabir, A. K. M. Anisur Rahman, Zeaur Rahim

**Affiliations:** 1 International Center for Diarrhoeal Disease Research, Dhaka, Bangladesh; 2 Department of Microbiology and Hygiene, Bangladesh Agricultural University, Mymensingh-2202, Bangladesh; 3 Department of Livestock Services, Ministry of Fisheries and Livestock, Dhaka-1215, Bangladesh; 4 Department of Microbiology, Jagannath University, Dhaka-1100, Bangladesh; 5 Department of Medicine, Bangladesh Agricultural University, Mymensingh-2202, Bangladesh

**Keywords:** Bayesian latent class analysis, farmed dairy cattle, gamma-interferon assay, single-intradermal comparative tuberculin test

## Abstract

**Background and Aim:**

Bovine tuberculosis (bTB) is an infectious disease of cattle, mainly caused by Mycobacterium bovis. This study aimed to evaluate the efficacy of the interferon-gamma (IFN-γ) assay and single-intradermal comparative tuberculin test (SICTT) in detecting bTB.

**Materials and Methods:**

In an earlier study, 150 positive, 83 inconclusive, and 480 negative animals from 24 cattle herds were screened using SICTT. From these groups, 125 positive, 17 inconclusive, and six negative animals were subsequently verified using the IFN-γ assay. Single-intradermal comparative tuberculin test outcomes were interpreted according to standard guidelines, whereas blood samples were collected and stimulated with purified protein derivatives. Sandwich enzyme-linked immunosorbent assay was used to measure secreted IFN-γ. Concordant and Bayesian latent class analyses were performed to evaluate test performance.

**Results:**

Results from the IFN-γ assay revealed that 83.2%, 64.7%, and 16.67% of the animals were positive in the SICTT-positive, inconclusive, and negative animal categories, respectively. Sensitivity (SE) and specificity (SP) of SICTT were 83.9% (95% confidence interval [CI]: 77.4–90.1) and 95.7% (95% CI: 86.9–99.7), respectively. Sensitivity and SP for the IFN-γ assay were 78.9% (95% CI: 71.9–85.4) and 83.9% (65.9–95.9), respectively. The use of both tests in parallel increases the SE of bTB detection (~94%), compared with SICTT alone.

**Conclusion:**

Use of the IFN-γ assay with SICTT in parallel, predominantly on cattle demonstrating an inconclusive SICTT outcome, boosts bTB detection rate in low resource settings.

## Introduction

Bovine tuberculosis (bTB) is a chronic wasting disease of bacterial origin, with ecological and economical significance for livestock and wildlife, as well as zoonotic implications. Typically, bTB is caused by *Mycobacterium bovis* infection; however, other species within the *Mycobacterium tuberculosis* complex (MTC) can be involved. In Bangladesh, there is limited active surveillance for estimating true MTC burden in cattle. Nevertheless, a few cross-sectional surveys have reported 8%–27% prevalence in crossbred cattle using the standard tuberculin skin test (TST) [1–4]. These magnitudes are used to notify the World Organization for Animal Health (WOAH) [5]. Further studies are needed to understand the prevalence and patterns of MTC infections in dairy cattle in Bangladesh.

Bovine tuberculosis is confirmed using two main approaches. The first focuses on identifying bTB-causing bacteria using techniques such as acid-fast bacilli staining and organ microscopy, bacterial isolation, histopathology, and antigen detection. The second approach involves detecting the immune response of the infected animal using delayed hypersensitivity skin tests, such as TST or blood-based laboratory tests, such as the interferon-gamma (IFN-γ) release assay — specifically the Bovigam assay. Other blood-based tests such as enzyme-linked immunosorbent assay (ELISA) and lateral flow antibody test can be used to detect the presence of antibodies against bTB [6]. However, TST is recommended for animal importation globally. Tuberculin skin test is used as single-intradermal comparative tuberculin test (SICTT) in European countries and as caudal fold tuberculin (CFT) test in New Zealand, North America, Australia, etc. [7]. Single-intradermal comparative tuberculin test distinguishes a delayed-type hypersensitivity reaction to intradermal inoculation of purified protein derivatives (PPDs), both bovine (PPDb) and avian (PPDa). In accordance with the directives of the WOAH (founded as OIE), CFT followed by comparative cervical skin test is used to identify bTB in dairy cattle, especially in government dairy farms, and to a lesser extent in private dairy farms. The directives are also used for animal trade in Bangladesh. However, antemortem bTB diagnosis in cattle is challenging due to cost and technical requirements. Therefore, most control programs focus on periodic screening and elimination of reactor animals from the herd using intradermal TST as the primary assessment tool [8]. Although not a definitive test for infection, TST has been used worldwide for decades as the primary first-line diagnostic test for identifying animals and humans with infection. However, like SICTT, TST has issues with sensitivity (SE) and specificity (SP) [9], and thus, alternative approaches, such as the Bovigam assay, are routinely used.

Purified protein derivatives used in SICTT are also used in a standard commercially available assay, that is, Bovigam (BOVIGAM, Prionics Lelystad BV, The Netherlands), for diagnosis of bTB in cattle [10]. Bovigam is a cell-based assay that uses the cell-mediated IFN-γ response to PPDb. Since 1988, this assay has been widely used in >200,000 cattle in the USA, Romania, New Zealand, Australia, Brazil, Spain, Ireland, Italy, etc. Sensitivity was 81.8%–100% and SP 94%–100% with culture-confirmed bTB [11]. Due to the cost and composite nature, laboratory-based assays like Bovigam are employed as ancillary tests or as test to confirm or nullify the results of TST/SICTT (serial testing) [6]. Thus, many countries have accepted the Bovigam assay as an official confirmatory test [12].

The success of a bTB eradication program depends on promptly confirming and eliminating reactors from a farm. However, the performance of SICTT and Bovigam assay in detecting bTB has not been evaluated in Bangladesh. The performance of any diagnostic assay, such as SE and SP, is usually evaluated by comparing it with a gold standard test. This includes isolating the pathogen and performing molecular and immunological assays. This approach to identify lesions and isolate the pathogen for bTB detection is impractical in areas lacking active abattoir surveillance. Alternatively, a Bayesian latent class analysis can be used to assess the performance of ≥2 bTB screening tests when true disease status is unknown [13]. In addition, Bayesian latent class analysis of the diagnostic test allows estimation of disease prevalence. However, none of the studies discussed used the Bovigam assay as a diagnostic test in Bangladesh.

This study aimed to assess the effectiveness of the IFN-γ assay and SICTT as diagnostic methods for bTB in dairy cattle from urban and peri-urban locations of Dhaka city, Bangladesh.

## Materials and Methods

### Ethical approval

The research was conducted at the International Center for Diarrheal Disease and Research, Bangladesh (icddr,b), and obtained approval from both the Research Review Committee and Ethical Review Committee. Furthermore, it was approved by the Animal Welfare and Experimentation Ethical Committee (AWEEC) of Bangladesh Agricultural University (BAU) (AWEEC/BAU/2019/24). Before collecting blood samples, written consent was obtained from the respective cattle farm owners/managers, and every effort was made to handle the animals in a humane manner to minimize stress, distress, discomfort, and pain during the sampling process.

### Study period and location

This cross-sectional survey was conducted from July to December 2019 among dairy cattle herds in urban and peri-urban settings of Dhaka City Corporation area. Initially, a total of 52 herds were chosen for the SICTT study, and further these herds were enrolled for the IFN-γ assay based on specific criteria.

### Inclusion and exclusion criteria for herd selection

**Figure-1:**
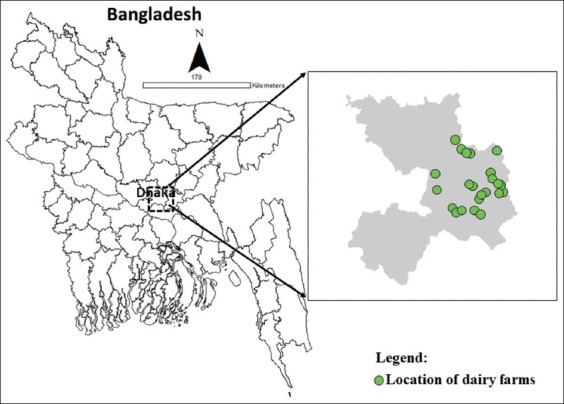
Map of study locations in Dhaka districts of Bangladesh. A total of 24 herds (farms) were enrolled under this study (as the coordinates of a few farms located closely could not be demonstrated independently) [Source: ArcGIS-ArcMap version 10.3].

Herds considered potential bTB reactors, with at least 3 SICTT-positive animals detected in each herd, were included in this study. Herds from which sample collection and dispatch to the designated laboratory could be accomplished within 6 h were included in this study. Based on inclusion and exclusion criteria, 24 herds were enrolled (Figure-1).

### Single-intradermal comparative tuberculin test

The SICTT was conducted by a team including a veterinarian and two paraprofessionals who were trained to conduct the test in cattle. In SICTT, both PPDb and PPDa (Prionics Leylastad BV) were injected intradermally following standard testing procedures [6]. On day 0, two 6–8 cm^2^ patches of skin were clean-shaven at 12 cm on the left side of the neck area of the test cattle. Standard slide calipers (Bar McLintock, Glasgow) were used to measure the size of skin induration of each shaved area. Then, 0.1 mL PPDb (3000 IU/mL) and 0.1 mL PPDa (2500 IU/mL) were administered intradermally in the two shaved areas using separate McLintock syringes (Bar McLintock).

Skin induration was further estimated at inoculation locations on day 3 (after 72 ± 6 h). Animals were considered (1) positive (reactor) if the rise in skin induration at PPDb was 4 mm more than that at PPDa; (2) inconclusive if the rise in skin induration between PPDb and PPDa was >0 and ≤4 mm; and (3) negative when the rise in skin induration at PPDb was ≤0 mm compared with that at PPDa [6].

### Gamma-interferon assay

The IFN-γ assay was performed using *M. bovis* IFN-γ test kit for cattle (BOVIGAM, Prionics Lelystad BV) [14].

### Collection of blood samples from cattle

Blood samples (4 mL) were collected in lithium heparin tubes from animals in positive, inconclusive, and negative groups after 3–30 d of SICTT and dispatched to the designated laboratory within 6 h of collection. The blood collection tubes were labeled with unique identifying numbers, and the samples were blinded for the IFN-γ assay.

### Blood stimulation and harvesting of plasma

In total, 375 μL of heparinized whole blood was aliquoted in triplicate in a sterile 96-well tissue culture plate. Blood cells were stimulated with 25 μL of nil control antigen (phosphate-buffered saline, pH-7.2), 25 μL PPDb (300 IU/mL), or 25 μL PPDa (250 IU/mL). Culture plates were incubated at 37°C under humidified conditions for 16 h for IFN-γ production and centrifuged at 500× *g* for 10 min to harvest 100 μL of plasma from each well.

### Bovine IFN-γ ELISA

Sandwich ELISA was used to assess IFN-γ production in stimulated plasma samples. An ELISA reader was used to measure absorbance with a 450 nm filter; a 620 nm filter was used as a reference (Dynex, Magellan Biosciences, USA). This assessment was performed in duplicate, and mean optical density (OD) values were calculated according to the instructions of the manufacturer (Bovigam, Prionics Lelystad BV). Positive and negative controls (supplied with the kit) were included in every test batch. Variation in OD was minor between batches. When OD PPDb−OD nil antigen was ≥0.1 and OD PPDb−OD PPDa was ≥0.1; OD PPDb−OD nil antigen was <0.1 or OD PPDb−OD PPDa was <0.1, the cattle were considered positive and negative, respectively.

### Data collection, management, and statistical analysis

Data for both SICTT and IFN-γ tests were entered in Microsoft Office Excel 2010 datasheet, exported to STATA 13 (StataCorp, College Station, Texas 77845, USA), and analyzed for descriptive and Cohen’s kappa statistics (interpreter reliability). Proportion, percentage, and 95% confidence interval (CI) were estimated for descriptive statistics.

### Concordant analysis

The reliability of the two tests was measured through concordance analysis through a descriptive graphical method, such as a point-cloud plot along the line agreement, and an analytical method for categorical variables using Cohen’s kappa statistics [15]. As per Cohen, the kappa test result was inferred as the degree of agreement, that is, none: ≤0; none-to-slight: 0.01–0.20; fair: 0.21–0.40; moderate: 0.41–0.60; substantial: 0.61–0.80; and almost perfect: 0.81–1.00, where p = 0.05 was considered statistically significant [16].

### Bayesian evaluation of the performance of the two tests

A Bayesian model for two conditionally dependent assays was used in a single population to estimate SE and SP of each assay [13]. The two tests were considered not dependent if the SE or SP of one assay did not influence the outcomes of the other assay. Because SICTT and IFN-γ have indistinguishable biological outcomes, their results can be interpreted as conditionally codependent [17]. The SE and SP of the assays under consideration were estimated based on their cross-classified outcomes in Bayesian latent class models. The SE and SP correlation coefficients between the two tests were estimated. Data relating to the SE and SP of SICTT and IFN-γ was obtained from other studies [18–21]. Beta distributions for SE and SP of both tests were estimated using the “findbeta” function of the PriorGen Package (https://cran.r-project.org/package=PriorGen) [22] in R 4.0.2 (https://www.r-project.org/) [23].

A Bayesian model was run in OpenBUGS (https://tinyurl.com/48ubv2j9) [24] through a burn-in period of 50,000 iterations and estimates based on an additional 50,000 iterations using three chains. Model convergence was assessed using time-series plots, autocorrelation plots, Gelman–Rubin convergence diagnostics, and Monte Carlo standard errors [25]. Three alternative priors were used to estimate the influence of priors on posterior estimates [26]. The OpenBUGS model code, including step-by-step explanations, was used.

## Results

### Demographic characteristics of the studied cattle

The median size of the dairy cattle herd was 51, with an interquartile range of 23–89. Most of the cattle were female (93.2%). Among all cattle, 61.5% (n = 91) were aged between 3 and 6 years, while 65% (n = 96) had a good (>6) body condition scores (Table-1).

**Table-1:** Summary of cattle (n = 148) included under Gamma-interferon assay (Bovigam^®^) from 24 dairy herds in urban and adjacent areas of Dhaka city, Bangladesh.

Parameter	Category	Frequency	%
Age	6–12 months	8	5.4
	1–3 years	23	15.5
	3–6 years	91	61.5
	>6 years	26	17.6
Sex	Male	10	6.8
	Female	138	93.2
Source of animal	Farm	84	56.8
	Bought	64	43.2
Breed	Frisian cross	138	93.2
	Local breed	2	1.4
	Jersey cross	4	2.7
	Sahiwal cross	4	2.7
Pregnancy status (n = 110)	Pregnant	73	66.4
	Non-pregnant	37	33.6
Body condition score	Good (>6)	96	64.9
	Medium (4–6)	48	32.4
	Bad (0–3)	4	2.7

### Comparative results between the two tests

To confirm the accuracy of SICTT, 148 animals (125 positive, 17 inconclusive, and nine negative) were tested using the INF-g assay. Of the 125 SICTT-positive animals, 104 (83.2%) were confirmed positive by the INF-γ assay. Of the 17 inconclusive animals, 11 (64.7%) were positive by INF-γ, and of the six negative animals, 1 (16.7%) was positive with the INF-γ assay (Table-2) [6].

**Table-2:** Comparative interpretation of SICTT (positive, negative, and inconclusive) and IFN-γ (positive and negative) test results as per standard criteria of the World Organization for Animal Health [6].

SICTT status	IFN- γ assay status		Total

	Positive (%)	Negative (%)	
Inconclusive (2–4 mm)	11 (64.70)	6 (35.30)	17
Negative (<2 mm)	1 (16.67)	5 (83.33)	6
Positive (>4 mm)	104 (83.2)	21 (16.80)	125
Total	116 (78.38)	32 (21.62)	148

SICTT=Single intradermal comparative tuberculin test, IFN-γ=Interferon-gamma

**Figure-2:**
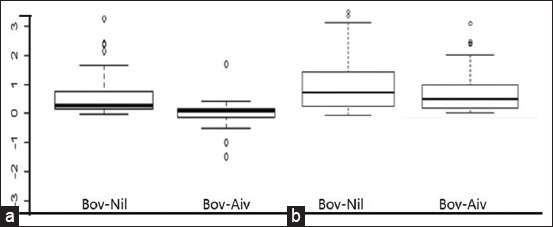
Comparison of the optical density (OD) values both in Bovine purified protein derivative (PPD)-Nil antigen and Bovine and Avian antigens after whole night culture (a) Bovigam^®^ Negative animals (n = 32); (b) Bovigam^®^ positive animals (n = 116) with the criteria of OD value of bovine PPD - nil antigen ≥0.1 and OD value of bovine PPD - avian PPD ≥0.1; and OD value of bovine PPD - nil antigen <0.1 or OD value of bovine PPD - avian PPD <0.1 were interpreted as positive and negative, respectively.

The stimulation and release of IFN-γ were higher after PPDa than PPDb treatment. Although the OD value of PPDb-nil antigen was ≥0.1, the OD value of PPDb–PPDa was not ≥1, which did not fulfill the standard criteria for a positive test (Figure-2a). However, OD PPDa compared to OD PPDb showed a difference of ≥0.1. The difference of OD values between PPDa and nil antigen of ≥0.1 indicated a positive result in IFN-γ positive animals (n = 116) (Figure-2b).

### Concordance analysis

#### Cohen’s kappa statistics

The agreement between the IFN-γ assay and SICTT was 77.7% (95% CI: 70.9%–84.5%). Therefore, the agreement obtained between the two tests is substantial and statistically significant (p = 0.004) (Table-3).

**Table-3:** Agreement obtained between SICTT and IFN-γ assay (Bovigam^®^) methods.

Interpretation of SICTT^1^	Interpretation of IFN- γ ^2^assay (Bovigam^®^)		

	Negative	Positive	Row total
Negative	11 (34.4%)	12 (10.3%)	23
Positive	21 (65.6%)	104 (89.7%)	125
Colum total	32	116	148
Kappa agreement rate	95% CI^3^	p-value	
77.7%	70.9%–84.5%	0.004	

1 SICTT=Single intradermal comparative tuberculin test,

2 IFN-γ=Interferon-gamma,

3 CI=Confidence Interval

#### Point-cloud plots

**Figure-3:**
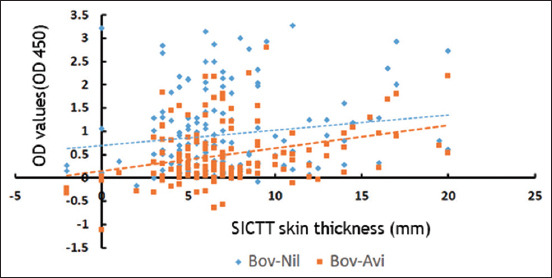
Comparison between two tests: Single intradermal comparative tuberculin test and gamma-interferon assay. The distribution of data points around the lines of agreement for these two tests is illustrated using a point-cloud diagram. The Y-axis on the diagram represents optic density values, specifically Bov-Nil and Bov-Aiv, and the X-axis represents the variation in skin thickness (mm) between bovine and avian sites following inoculation with purified protein derivatives.

The difference in skin thickness in SICTT (bovine minus avian sites) (mm) in relation to difference in OD (Bov-Nil and Bov-Aiv) was presented graphically, using point-cloud plots. The point-cloud plots are significantly distributed near the agreement lines (Figure-3).

#### Sensitivity and SP of SICTT and IFN-γ assay

Cross-classified test results for SICTT and IFN-γ are presented in Table-4 [6]. Approximately 69% of the samples tested positive with both tests. The SE and SP of SICTT were 83.9% (95% CI: 77.4–90.1) and 95.7% (95% CI: 86.9–99.7), respectively. The SE and SP of the IFN-γ assay were 78.9% (95% CI: 71.9–85.4) and 83.9% (65.9–95.9), respectively. Sensitivity increased to 93.8% (95% CI: 88.6–97.6) when a parallel interpretation was used (an animal is considered positive if it is found positive in at least one test) for SICTT and IFN-γ assay.

**Table-4:** Cross-classified results of SICTT and IFN-γ assay in the studied sample (inconclusive interpretation of SICTT were considered as negative).

Tests		Number of animals (%)

SICTT^1^	IFN- γ assay^2^	
Positive	Positive	102 (68.9)
Positive	Negative	22 (14.9)
Negative	Positive	13 (8.8)
Negative	Negative	11 (7.4)
Total		148 (100)

1 Parameter for SICTT-positive: Skin thinness difference among bovine and avian sides at >4 mm as per standard criteria [6],

2 IFN-γ ELISA as per criteria, the OD values of bovine PPD-nil antigen ≥0.1 and OD values of bovine PPD-avian PPD ≥0.1; and OD values of bovine PPD-nil antigen <0.1 or OD values of bovine PPD-avian PPD <0.1 were interpreted as positive and negative, respectively, SICTT=Single intradermal comparative tuberculin test, IFN-γ=Gamma-interferon assay, PPD=Purified protein derivative

By contrast, SP increased to 97.6% (95% CI: 91.7–99.9) when serial interpretation was used (an animal is considered positive if it is found positive in both assays) for SICTT and IFN-γ assay. The correlation coefficient between SICTT and IFN-γ assay in infected and healthy cattle was 18.7% (Table-5).

**Table-5:** Estimates of sensitivity and specificity of SICTT and IFN-γ assay based on Bayesian model.

Tests	Sensitivity (95% Cr. I.^1^)	Specificity (95% Cr. I.)
SICTT	83.9 (77.4–90.1)	95.7 (86.9–99.7)
IFN-γ	78.9 (71.9–85.4)	83.9 (65.9–95.9)
Parallel interpretation between SICTT and IFN-γ	93.8 (88.6–97.6)	81.9 (64.2–94.3)
Serial interpretation between SICTT and IFN-γ	69.1 (61.6–76.4)	97.6 (91.7–99.9)

**Correlation coefficient between**	**Mean (95% Cr. I.)**	

Sensitivities of SICTT and IFN-γ	18.7 (1.5–39.6)	
Specificities of SICTT and IFN-γ	23.7 (0.8–71.4)	

Cr. I.=Credibility Interval, SICTT=Single intradermal comparative tuberculin test, IFN-γ=Gamma-interferon

Table-6 demonstrates the results of SE analyses. Posterior estimate changes in SE of both tests were not significant (change >25% of median value) when non-informative distributions were employed as priors for any parameter representing model robustness.

**Table-6:** Sensitivity and specificity estimates under alternative prior specifications (sensitivity analysis).

Tests and models	Sensitivity (95% Cr. I.)	Sensitivity (95% Cr. I.)
Uniform priors in the range of 0–1 for SEs and SPs		
SICTT	74.5 (12.5–98.1)	24.9 (1.7–88.9)
IFN-γ	69.6 (10.7–97.2)	29.7 (2.8–87.9)
Uniform priors in the range of 0–1 for SPs and the informative priors for SEs used for the primary analysis		
SICTT	76.7 (57.2–88.4)	19.3 (0.8–80.6)
IFN-γ	79.9 (65.2–91.8)	35.8 (4.4–92.5)
Uniform priors in the range of 0–1 for SEs and the informative priors for SPs used for the primary analysis		
SICTT	87.3 (79.5–95.3)	95.6 (87.2–99.7)
IFN-γ	79.5 (71.9–95.3)	82.5 (62.7–95.7)

Cr. I=Credibility Interval, SICTT=Single intradermal comparative tuberculin test, IFN-γ=Gamma-interferon, SE=Sensitivity, SP=Sensitivity

However, substantial changes were observed in posterior estimates for SP of SICTT (95.7%–24.9%) and IFN-γ assay (from 83.9% to 29.7%), These changes underscore the substantial impact of the prior information on specificity within the model.

## Discussion

To the best of our knowledge, this study is the first to link the IFN-γ assay with SICTT in commercial dairy cattle in Bangladesh. Based on our results, we recommend the parallel use of SICTT and IFN-γ assay to enhance the overall detection rate of bTB in an infected cattle herd and reduce SICTT false positives. This study demonstrated the near-equivalence of two screening techniques through concordance analysis. Cohen’s kappa (*k*) showed 77.70% agreement (95% CI: 70.9%–84.5%, p = 0.004) between the two tests, which was statistically significant. This agreement was comparable with a study conducted in Korea using the single CFT skin test and commercial interferon assays: CFT versus ID screen ELISA (ID-Vet) 87%; and CFT versus TB-Feron ELISA (Bionote) 91% [27]. A study in Ethiopia demonstrated similar performance of TST and ID Screen ELISA to detect bTB in zebu cattle [28]. Of the 125 SICTT-positive animals in our study, 104 (83.3%) also tested positive with the IFN-γ assay. However, only one-half (n = 11, 47.8%) of the animals were confirmed as negative among SICTT-negative animals (n = 23) using this ancillary test.

In this study, the SE of SICTT was 83.4%, similar to that reported by Praud *et al*. [29]. The SE of the INF-γ assay was 78.9%, which was comparable with similar studies [19, 30]. Consistently, the SE of the INF-γ assay reportedly varied from 80.9% to 100% [30]. Van der Heijden *et al*. [30] found that Bovigam showed the highest SE (100%, 8/8) and good agreement with SICTT for the detection of bTB in African buffaloes. Conversely, lower SE estimates for the INF-γ assay (60.1%) were reported in 128 milking cows from 25 bTB-infected herds by Singhla *et al*. [13] in Thailand.

Interestingly, 17% (21/125) of SICTT-positive animals tested negative with the IFN-γ assay (Bovigam) (Table-2). Under dual infection conditions (*M. bovis* and *Mycobacterium avium* subsp. *paratuberculosis* [MAP] or environmental *Mycobacterium* species), the immune response established in the animals may reduce the SP of diagnostic assays designed to measure targeted response against each pathogen. Dissimilarities in the percentage of *M. bovis-*infected cattle responding to the IFN-γ assay were detected due to the presence of MAP/environmental *Mycobacterium* in animals, where both infections were more likely to show false negative outcomes. This point focuses on the presence of MAP/environmental *Mycobacterium* species in a herd when conducting the IFN-γ assay, which not only interferes with SP, but also lowers SE, with 50% and 78.3% in herds with MAP coinfection and animals infected with bTB, respectively [31]. Because MAP infection decreases the SP of bTB diagnostic tests, studies must interpret the contribution of coinfections in the diagnosis of bTB. Exposure to coinfections can significantly influence the outcomes when interpreting IFN-γ assay results [32].

In addition, our study confirmed that 64.7% of inconclusive animals with SICTT (n = 17) were positive with the IFN-γ assay (Table-2). Although SICTT is used globally, age, body condition score, medication, and parturition, immune status, etc., could influence the precision of test outcomes [33]. Some animals in the inconclusive group could have been genetically poor responders to SICTT or in an advanced state of disease progression and developed an anergic condition [34]. The judicious application of IFN-γ assay combined with SICTT can be used to detect *M. bovis-*infected cattle in bTB breakdown farms [10, 34, 35]. In an infected herd, the IFN-γ test can identify extra-risk animals that might cause future outbreaks [12, 36]. A Cox-proportional hazard model study [12] showed that animals tested positive with the IFN-γ assay were >2 times more likely to develop bTB than IFN-γ negative animals. Other studies have confirmed this finding [33–35, 37].

In this study, a single SICTT-negative animal was found to be IFN-γ positive. Consistently, SICTT-negative animals showing IFN-γ positivity had a 7–9 times greater odds of becoming SICTT-positive in a future test [34, 35].

In this study, 52% (12/23) of additional bTB positivity in animals was identified using the IFN-γ test, whereas SICTT excluded these animals as inconclusive or negative. The IFN-γ assay has high SE and can be used as a novel benchmark for bTB screening [38, 39]. Therefore, the INF-γ test should be included in the bTB eradication program as an ancillary test to TST to enhance SE, facilitate early detection, and reduce the chance of false negative SICTT results. In this study, approximately ~17% (21/125) of tested cattle showed false negative results by the IFN-γ test, which can be avoided if there were criteria for the interpretation of INF-γ results with mixed or MAP infections or other mycobacterial infections. The use of SICTT and IFN-γ assay in parallel enhances the SE (~94%) of the screening program and is useful in identifying infected animals. Proper identification of infected animals will facilitate the control and eradication of bTB [40].

The study has some limitations. The study included a small number of cattle, and most test animals had positive or inconclusive outcomes according to SICTT. Thus, decreased SP estimates were expected for both SICTT and INF-γ assay. However, we observed higher SP for these tests, which was largely influenced by prior information we supplied. Hence, the SP estimates of the SICTT and INF-γ test should be interpreted with caution. Future studies with a representative sampling technique must be performed in farmed cattle.

## Conclusion

The IFN-γ assay (Bovigam) can be used for ancillary testing with SICTT to boost the screening of bTB infection in dairy cattle herds. In this study, the IFN-γ assay identified additional bTB-infected cattle that were considered inconclusive or negative with SICTT alone. False positive SICTT results can be affected by coinfection from animals with MAP or other *Mycobacterium* species. False positives can be ameliorated by the ancillary application of the IFN-γ test. Parallel testing (SICTT and IFN-γ assays) could enhance the overall detection of bTB in an infected herd and reduce SICTT false positives. In conclusion, the findings of this study emphasize the need for adjusting combined screening tests, particularly in highly infected cattle herds.

## Data Availability

All the data, including the information presented in the main text, tables, and figures, are provided in the manuscript.

## Authors’ Contributions

SSI, RI, and MMHF: Contributed to the SICTT, and subsequent blood sample collection from animals. TBR, SSI, ZR, SMLK, and RI: Performed laboratory assessment and wrote a draft manuscript. AKMAR: Performed statistical analysis of the data and reviewed the manuscript. All authors have read, reviewed, and approved the final manuscript.
